# Correspondence

**Published:** 1876-04

**Authors:** 

**Affiliations:** Port Huron, Mich.


### Correspondence.

PORT HURON, MICH., Feb. 23, 1876.

TO J. TAFT, EDITOR:

In the case of the Goodyear Dental Vulcanite Co., *vs.* Dr. Kibbee of this place, argued before Hon. Judge H. H. Emmons at Detroit, a few days since, in which the Company and Josiah Bacon, their agent, claim that the use of celluloid for dental plates is covered by the Cummins patent, were some points the dentists and their patrons would like to have explained. Some time last year the Vulcanite Co. commenced suit against E. M. Flagg of New York, and on the hearing Judge Blatchford said "it is not sufficiently clear that this process (meaning celluloid) is embraced in the claim of the plantiff's patent to warrant the granting of an injunction etc.," Bacon then with drew the suit and paid the cost. The company by Bacon then come to Michigan and to this city and commenccd suit against Dr. Kibbee, while several dentists in the city of Detroit, where the court is held, are using celluloid, as Bacon and his attorney are fully aware. And on the morning of the hearing an article appeared in one of the Detroit papers stating it was a test case transferred from the New York court on account of the crowded state of the dockets there, and the first that had been in the courts, also that Mr. Ashly was for the plantiff and B. R. Lee for defence, whereas, both Ashly and Lee are plantiff's attorneys, and no one appeared for the defence. The whole thing was calculated to deceive.

I understand the question was asked why they did not take the test case to Massachusetts, near where both the Vulcanite and Celluloid Companies are located? The answer was that rumor said the judges were biased. Now we would like to know,

1st. Why suit was not commenced against some one of the dentists in Detroit, instead of making Dr. Kibbee the trouble of going sixty or seventy miles and back to give his testimony ?

2d. Why was the trial kept so secret that the dentists in Detroit did not know of it until the morning of the hearing ?

3d. Why it was necessary for plantiffs to bring suit so far away from the office of the Celluloid Co. who agreed to defend those who use their plates. When there are plenty of cases to be found nearer where both companies are located, outside of New York city and Massachusetts?

4th. Why the Celluloid Co. did not send some one there to defend the case if they had notice in time, as the plantiffs say they did and was urged to come?

It is also understood by some that last fall while the case of the Dental Vulcanite Co. *vs.* Willis was in progress in Detroit, that Josiah Bacon and one of his attorneys were entertained by Judge Emmons at his house in Ecorse. I could hardly believe it. But in these days of crooked whisky, etc. it will do no harm to enquire for those without reproach, do not fear to have their acts investigated and if there is wrong, the sooner it is brought to light the better for community.

1. Did Mr. Lee or Ashly or Bacon stay with judge Emmons at his house over night during the Willis trial?

2. Did Bacon or Lee or either one of them stay over Sunday with the judge and help write his decision, or write it at his dictation?

3. Did Bacon obtain a copy then?

4. Was it not the treatment that Bacon and his counsel received here at that time, that encouraged them to make the effort here to get the start of the Celluloid Co. and scare the dentists into taking out another year's license for the use of the rubber?

Those interested and knowing the facts, please answer.

JUSTICE.

